# Sleep quality, coping, and related depression: A cross‐sectional study of Turkish nurses

**DOI:** 10.1111/inr.12928

**Published:** 2024-01-19

**Authors:** Gülseda Topal Kılıncarslan, Ayşegül Özcan Algül, Nalan Gördeles Beşer

**Affiliations:** ^1^ Niğde Ömer Halisdemir Training and Research Hospital Niğde Turkey; ^2^ Department of Publıc Health Nursing Nursing Department Semra and Vefa Küçük Faculty of Health Sciences, Nevşehir Hacı Bektaş Veli University Nevşehir Turkey; ^3^ Department of Psychiatric Nursing Nursing Department Niğde Zübeyde Hanım School of Health, Niğde Ömer Halisdemir University Niğde Turkey

**Keywords:** Depression, nursing, sleep quality, stress coping strategies

## Abstract

**Background:**

The sleep quality of nurses affects both their health and standard of nursing care. Working conditions, depression, and coping potential can also lead to sleep problems.

**Introduction:**

Sleep plays a crucial role in overall health at every stage of life. The purpose of this study is to determine the effect of sleep quality, depression, and coping mechanisms on the performance of nurses, whose sleep quality is strongly affected due to shift‐based work.

**Methods:**

The sample of this descriptive correlational study consisted of 133 healthcare workers. Data were collected using the Personal Information Form, Beck Depression Inventory (BDI), Coping Response Inventory (CRI), Pittsburgh Sleep Quality Index (PSQI), and Epworth Sleepiness Scale (ESS) and evaluated using the *t* test, Mann–Whitney *U* test, one‐way ANOVA, Enter method, and linear regression.

**Findings:**

One in three nurses reported having poor‐quality sleep, and one in two nurses said they felt sleepy during the day. With declining sleep quality, the nurses' ability to cope with stress diminished, and their degree of depression increased.

**Discussion:**

The nurses' financial situation and level of depression were key factors that influenced their quality of sleep and capacity to handle stress. To improve nurses' sleep, the shift pattern needs to change.

**Conclusion:**

To increase the quantity of sleep and enhance mental health, changes should be made to the shift schedules of nurses to allow for appropriate rest and reduce daytime sleepiness.

**Implications for nursing practice and policies:**

Improved working conditions for nurses and updated nursing standards are required to improve nurses health and wellbeng.

## INTRODUCTION

The physical, social, and mental health of adults is affected by sleep (Matricciani et al., 2017). Health professionals who work on shifts experience sleep issues and a disruption in their circadian rhythms which physiologically control the sleep–wake cycle and prepare the body for night sleep (Bumin et al., 2019; Cable et al., 2021). In Turkey, the “health industry” (“healthcare” hereafter) accounts for 40% of all shift work (Eurofound, 2019). As part of their duty to offer round‐the‐clock patient care in hospitals, nurses invariably work shift schedules (Şanlı & Yürümezoğlu, 2020). The primary causes of sleep issues in nurses are night shifts and long shifts (Bumin et al., 2019). In a meta‐analysis of studies on the sleep quality of nurses, 61% of the nurses were found to have low‐quality sleep (Zeng et al., 2020). Zeng et al. (2020) observed that 61% of the nurses showed symptoms of poor sleep quality, but Dong et al. (2020) indicated that more than 65.8% of nurses faced issues such as insufficient sleep. Similar findings were reported for Turkish nurses, whose sleep quality ranged from 50.5% to 97.8% (Pirinçci et al., 2021). Sleep issues faced by nurses also contribute to problems in focus, long‐term health issues, and workplace accidents (Ganesan et al., 2019; Karahan et al., 2020).

In the literature, factors such as shift work, caffeine consumption, work–family conflict, anxiety, and stress have been reported to affect sleep quality negatively (Chen et al., 2017; Liu et al., 2020). According to the International Labor Organization, one of the primary sources of stress in the workplace for nurses is the shift system and alternating day and night shifts (Leso et al., 2021). According to their research on Turkish nurses, the most significant sources of stress were shift work, low pay, and exposure to workplace hazards that could endanger one's health (Küçük & Yağmur, 2018). As per the recommendations of the European Union countries, the International Labour Organization, and the International Council of Nurses (ICN), adjustments have been made to the working and resting hours of nurses. In Turkey, night shifts for nurses are defined as “*nöbet*.” The night shift hours are implemented in a two‐shift system (0800−0800 and 1600−0800) or a three‐shift system (0800−1600, 16.00−0000, 0000−0800). Additionally, although the designated weekly working hours for nurses are 45 hours, it is known that nurses often work beyond this duration (Şanlı & Yürümezoğlu, 2020).

Nurses must be in a state of complete well‐being in order to guarantee the standard of care (Kesgin & Çağlar, 2020). Understanding nurses’ quality of sleep and the factors that influence, it is believed to be helpful for improving patient care and safeguarding the nurses' personal health (Küçük & Yağmur, 2018; Pirinçci et al., 2021). In a systematic review, it was reported that shift work causes sleep problems and fatigue, negatively impacting the health of nurses and patient safety and care. However, there is no strong evidence showing the effects of sleep and the circadian rhythm on fatigue in nursing shift work (Querstret et al., 2020). Numerous studies on the risks faced by nurses worldwide (Chen et al., 2017; Liu et al., 2020; Zeng et al., 2020) and in Turkey (Akçay et al., 2021) have been published in the literature, but few very studies have examined stress, sadness, and risk factors for poor sleep quality among nurses (Chen et al., 2017; Küçük & Yağmur, 2018). Therefore, this study aimed to determine the quality of sleep of nurses and the influencing factors.

The following research questions were addressed:

1. How well nurses sleep?
2. Does the use of stress coping strategies by nurses affect the level of depression they experience?
3. Does depression and use of coping stratagies impact the sleep quality of nurses?

## METHODS

### Design

This study aimed to determine the sleep quality of nurses and factors that affect it. This was a descriptive study that followed the STROBE checklist.

### Sample and setting

In all, 201 nurses working at a hospital in Turkey were considered eligible for this study. The final sample consisted of 133 nurses who voluntarily agreed to participate in the study between January and May 2019. The sample size of 133 participants was based on an effect width of 0.426 and a significance threshold of 0.05, at a power of 99.99%. G*Power 3.1.9.7 was used as part of the power analysis process.

We aimed to reach the entire population, and 66.2% of it was reached. Nurses who participated in the pre‐application (*n* = 15), those who were on leave without pay, those who had a doctor's report, those who were not in the institution during the research period (*n* = 22), and those who did not agree to participate in the study (*n* = 31) were excluded.

After informing the nurses about the study and obtaining their verbal consent, the researchers conducted face‐to‐face interview to collect the data. Data were collected using a data collection form, based on the results of our literature review, and other relevant scales, listed below. The nurses were given a time frame to return the completed questionnaire; the form took approximately 10−15 minutes to complete.

### Data collection tools

The sociodemographic data of the nurses were collected using a personal information form; Beck's Depression Inventory (BDI), Coping Responses Inventory (CRI), Pittsburgh Sleep Quality Index (PSQI), and Epworth Sleepiness Scale were used to collect additional data.

### Personal information form

The personal information form, designed by the researcher after reviewing the literature, comprised 16 questions on the sociodemographic details of the participants and personal traits that are thought to influence sleep quality.

### Beck's depression inventory

Developed by Beck (1961), BDI is used to determine the risk of depression and to measure the level and severity of depression symptoms. It consists of 21 items (Beck et al, 1961). The highest score that can be obtained in the BDI is 63, and the lowest score is 0. Scores of 17 and above are defined as the presence of depressive symptoms. The validity and reliability of the Turkish version were tested by Hisli, 1989. In our study, the Cronbach's alpha was 0.854.

### Coping responses inventory

Developed by Moos in 1993, the CRI is a self‐assessment scale that examines the coping mechanisms applied to stressful life experiences. The scale consists of eight variables and is divided into two parts: approach coping strategies and avoidance coping strategies. The section on approach coping strategies includes the dimensions of logical analysis, positive reevaluation, seeking guidance and support, and problem‐solving, and the section on avoidance coping strategies includes the dimensions of cognitive avoidance, acceptance or resignation, creating alternative rewards, and emotional discharge (Moos, 1993). Participants were asked to answer the questions by thinking about the most stressful event they had encountered in the last 12 months. The reliability and validity of the scale in the context of Turkey were tested by Ballı and Kılıç (2016). Given the scope of the study, only one part of the scale (approach responses) and four dimensions (logical analysis, positive evaluation, seeking guidance and support, and problem‐solving) were used. The internal consistency coefficients, denoted by Cronbach's alpha, ranged from 0.73 to 0.91 (Ballı & Kılıç, 2016). High scores indicated higher levels of coping strategies for dealing with stress. In our study, the Cronbach's alpha was 0.940.

### Pittsburgh sleep quality index

Developed by Buysse et al. (1989), the PSQI is a self‐report screening and measurement scale that collects detailed information on sleep quality and the type and severity of sleep disorders, if any, within the last month (Buysse et al., 1989). It consists of 24 questions on seven components: subjective sleep quality, sleep latency, sleep duration, habitual sleep efficiency, sleep disturbances, use of sleeping medications, and daytime dysfunction. The scores on the seven components are summed to achieve the total PSQI score. The total PSQI score can range from 0 to 21. A total score of 5 or below denotes “good” sleep, and a total score above 5 denotes “poor” sleep, indicating the respondent is experiencing problems in at least two areas of sleep or mild‐to‐moderate distress in more than three areas. The validity and reliability of the Turkish version of this index were determined by Ağargün et al. (1996). In our study, the Cronbach's alpha was 0.793.

### Epworth sleepiness scale

Developed by Johns (1991), the ESS is a widely used scale that is easy to apply and evaluate (John, 1991). ESS consists of items scores on a four‐point Likert scale of 0, 1, 2, and 3; a high score indicates a high level of daytime sleepiness. It is a simple self‐report scale used to measure the general level of daytime sleepiness. The validity and reliability of the ESS in Turkey were determined by Ağargün et al. (1999). A total score greater than 10 indicates excessive daytime sleepiness. In our study, the Cronbach's alpha was 0.777.

### Data analysis

The data were analyzed using IBM SPSS V23. Normal distribution of data was confirmed using the Kolmogorov–Smirnov and Shapiro–Wilk tests. For the comparison of quantitative variables by the paired group method, an independent two‐sample *t* test was used for normally distributed data, and Mann–Whitney *U* test was used for nonnormally distributed data. When comparing quantitative variables according to groups of three or more, one‐way analysis of variance (ANOVA) was used for normally distributed data, and the Kruskal–Wallis test was used for nonnormally distributed data. Spearman's correlation coefficient was used to measure the relationship between nonnormally distributed scores. Linear regression analysis was used to analyze the independent variables affecting the PSQI values, and the Enter method was used to include the independent variables in the model. The results are presented as mean, standard deviation, and median (minimum–maximum) values for quantitative data and as frequency (*n*) and percentage (%) values for categorical data. The significance level was set at *p* < 0.05.

### Ethical approval

This study was approved by the Nevşehir University Non‐invasive Research Ethics Committee (Number: 2019.03.31). Institutional permission was also obtained from the nurses’ hospital, and participation was voluntary.

## FINDINGS

### Participants’ sociodemographic characteristics

In all, 88.7% of the participants were women, 58.6% were married, 26.3% currently smoked, 13.5% smoked 16−20 cigarettes a day, 85% did not consume alcoholic drinks, 95.5% consumed tea, and 68.4% consumed coffee. According to the PSQI, 39.8% of the nurses had poor sleep quality, and 25.6% had a long‐term sleep disorder. As per the ESS data, 42.1% of the nurses experienced daytime sleepiness, and 42.9% suffered from mild depression.

### Participants’ characteristics concerning sleep quality

The mean PSQI scores of the groups with different durations of experience were statistically and significantly different (*p* < 0.05) (Table 1). The mean PSQI score was 9.1 ± 4.4 (1−19) for those with 1−5 years of experience, 8.7 ± 4.7 (0−19) for those with 6−10 years of experience, and 7.3 ± 4.3 (1−18) for those with 11−20 years of experience. The mean PSQI score of nurses with a few years of experience was higher than that of nurses with many years of experience (*p* < 0.05) (Table 1).

**TABLE 1:** Distribution of Pittsburgh sleep quality index and Epworth sleepiness scale scores according to working characteristics and some habits of the nurses.

	Pittsburgh sleep quality index			Epworth sleepiness scale		
Working Characteristics	*x̄* ± *S.s*	Median (min.–max.)	*p*	*x̄* ± S.s	Median (min.–max.)	*p*
**Number of working years**						
1–5	9.1 ± 4.4	9 (2–19)^a^	$\chi^{2}$ = 6.412 0.041*	8.3 ± 4.8	8 (0–24)	*F* = 1.902 0.153
6–10	8.7 ± 4.7	8 (0–19)^ab^		9.3 ± 5.1	10 (0–18)	
11–20	7.3 ± 4.3	6 (1–18)^b^		7.1 ± 4.5	7 (0–18)	
**Clinic**						
Surgical unit, internal unit, and emergency department	9.2 ± 5.2	8 (0–19)	$\chi^{2}$ = 3.916 0.141	8.7 ± 4.9	8 (0–24)	*F* = 2.781 0.066
Intensive care	7.6 ± 3.2	8.5 (2–14)		7.9 ± 4.8	8 (0–18)	
Outpatient clinic, dialysis, and other	7.2 ± 3.9	6 (3–18)		6.4 ± 3.9	6 (0–17)	
**Position**						
Chief nurse	5.5 ± 2.7	5 (2–10)	*U* = 694.5 0.065	5.6 ± 4.4	4.5 (0–14)	*U* = 657.0 0.136
Nurse	8.4 ± 4.5	8 (0–19)		8 ± 4.7	8 (0–24)	
**Working in shifts status**						
Yes	8.6 ± 4.4	8 (0–19)	*U* = 614.5 0.006*	7.9 ± 4.7	8 (0–24)	*U* = 971.0 0.673
No	6.1 ± 4.5	5 (2–18)		7.5 ± 5.1	6.5 (2–18)	
**Number of shifts per month**						
1–5	7.8 ± 4.6	7 (0–19)^ab^	$\chi^{2}$= 7.011 0.030*	7 ± 4.5	6.5 (0–18)	$\chi^{2}$ = 5.448 0.066
6 and above	9 ± 4.1	9 (1–19)^a^		8.8 ± 4.8	9 (0–24)	
No shifts	6.6 ± 5	5 (2–18)^b^		7.1 ± 4.7	5.5 (2–14)	
**Coffee consumption status**						
Yes	8.7 ± 4.3	8 (0–19)	*U* = 1482.5 0.037*	8.1 ± 4.8	8 (0–24)	*U* = 1776.0 0.512
No	7.2 ± 4.6	6.5 (1–19)		7.5 ± 4.5	7.5 (0–18)	
**Tea consumption status**						
Yes	8.1 ± 4.4	7 (0–19)	*U* = 548.0 0.069	8.1 ± 4.6	8 (0–24)	*U* = 125.0 0.005*
No	11.5 ± 4.6	11 (5–18)		2.7 ± 3.4	1 (0–8)	

Abbreviations: $\bar{x}$, arithmetic mean; $\chi^{2}$, Kruskal–Wallis test statistic; ^ab^. There is no difference between cases with the same letter; *P*, test statistic; S.s, standard deviation; *U*, Mann–Whitney *U* test statistic. p < 0.05.

The scores of the nurses who did not work in shifts were significantly higher than the scores of those who worked in shifts. The mean PSQI scores of nurses in different shifts in a month were statistically and significantly different (*p* < 0.05) (Table 1). The mean scores of nurses with 1−5 shifts, 6 or more shifts, and no shifts were 7.8 ± 4.6 (0−19), 9 ± 4.1 (1−19), and 6.6 ± 5.0 (2−18), respectively. The average sleep quality score of the nurses who had no shifts was higher than that of the nurses with 6 or more shifts.

A statistically significant difference was found in the mean PSQI scores of the nurses who did and did not drink coffee (*p* < 0.05) (Table 1). The mean score of the nurses who drank coffee was 8.7 ± 4.3 (0−19) and the score of those who did not drink coffee was 7.2 ± 4.6 (1−19). The mean scores of the nurses who drank coffee were higher than the scores of those who did not.

### Relationship between sociodemographic variables, coping response inventory, and depression

The mean BDI scores of nurses in different economic situations were statistically and significantly different (*p* < 0.05; Table 2). The mean BDI score of nurses with moderate economic status was 14.4 ± 8.7 (1−46) and that of nurses with poor economic status was 18.8 ± 10 (9−36). Nurses with high economic status had lower mean BDI scores than those with poor economic status.

**TABLE 2:** Distribution of mean scores from Beck's depression inventory and coping responses inventory according to nurses' descriptive characteristics.

	Beck's depression inventory			Coping responses inventory		
Descriptive characteristics	x̄ ± S.s	Median (min.–max.)	*p*	*x̄* ± S.s	Median (min.–max.)	*p*
Female	12.9 ± 7.3	12 (1–36)	*U* = 813.5 0.610	86.9 ± 14.2	87 (24–120)	*U* = 825.5 0.672
Male	14.4 ± 13.7	11 (1–46)		82.7 ± 21.7	88 (24–115)	
**Educational status**						
Health vocational high school and associate degree	10.6 ± 6.1	11 (1–22)	*U* = 1631.0 0.173	87.9 ± 12.5	87.5 (69–115)	*U* = 1349.0 0.812
Undergraduate and graduate	13.7 ± 8.6	12 (1–46)		86.1 ± 15.7	87 (24–120)	
**Marital status**						
Married	11.5 ± 6.7	12 (1–46)	*U* = 2559.0 0.058	87 ± 15.3	87 (24–120)	*U* = 1943.5 0.357
Single	15.4 ± 9.6	14 (3–45)		85.7 ± 15	87 (55–120)	
**Economic status**						
Good	9.9 ± 5.7	10 (1–27)^a^	$\chi^{2}$ = 11.374 **0.003**	91.8 ± 14.4	92 (66–120)^a^	$\chi^{2}$ = 8.058 **0.018**
Average	14.4 ± 8.7	12 (1–46)^b^		84 ± 15.1	85.5 (24 −120)^a^	
Bad	18.8 ± 10	17.5(9‐36)^ab^		80.2 ± 9.5	81.5 (65–89)^ab^	
**Way of coming to work**						
Private car	11.6 ± 7.6	10 (1–45)	$\chi^{2}$ = 4.771 0.092	88.1 ± 16.7	87.5 (24–120)	*F* = 0.845 0.432
Bus	13.6 ± 9.7	12 (1–46)		83.9 ± 16.2	85 (24–120)	
By foot	14.4 ± 7.4	14 (2–36)		86.8 ± 12.1	88 (55–118)	
**Parental status**						
Yes	12.3 ± 8.3	12 (1–46)	*U* = 2435.0 0.313	86.9 ± 16.4	88 (24–120)	*U* = 1982.0 0.303
No	13.9 ± 8.2	12 (1 – 36)		86 ± 13.9	86(55–120)	

Abbreviations: $\bar{x}$, Arithmetic mean;$\chi^{2}$, Kruskal–Wallis test statistic. ^ab^, There is no difference between cases with the same letter; *P*, test statistic; S.s, standard deviation; *U*, Mann–Whitney *U* test statistic. p < 0.05.

The mean CRI scores of the nurses in different economic situations were statistically significantly different (*p* < 0.05) (Table 2). The average score for nurses in high financial standing was 91.8 ± 14.4 (66−120), that for nurses in moderate financial standing was 84 ± 15.1 (24−120), and that for nurses in poor financial standing was 80.2 ± 9.5 (65‐89). The CRI yielded the highest mean scores for nurses with high economic status and the lowest mean scores for nurses with low economic status.

The ESS scores of nurses who did and did not drink tea were statistically and significantly different (*p* < 0.05) (Table 2). The mean ESS score of nurses who drank tea was 8.1 ± 4.6 (0−24), while the average score of those who did not drink tea was 2.7 ± 3.4 (0−8). The nurses who never drank tea had lower median sleepiness scores than those who drank tea.

The mean PSQI scores of nurses in different age groups were statistically and significantly different (*p* < 0.05). The average age of those who slept well was 39 years, the average age of those who slept poorly was 30 ± 8.4 (20−52), and the average age of those who had long‐term sleep difficulties was 27.5. The mean scores of the ESS and BDI did not differ significantly among the age groups (*p* > 0.05).

The PSQI and BDI scores showed a statistically significant positive and moderate connection (*p* < 0.001; *r* = 0.426) (Table 3). In other words, the nurses' sleep quality declined as their level of depression increased. The CRI and BDI scores showed a statistically significant negative and moderate connection (*p* ± 0.001; *r* = −0.484) (Table 3). In other words, the nurses' degrees of depression declined as their use of coping mechanisms increased. In this study, a low‐level negative correlation was found between the CRI and ESS scores (*p* = 0.016; *r* = −0.209) (Table 3). In other words, the nurses' use of stress‐reduction techniques declined as their level of tiredness increased. Other scale scores did not show any statistically significant correlations (*p* > 0.05) (Table 3).

**TABLE 3:** Distribution of the relationship between scale scores.

Scales		PSQI	BDI total	CRI total	ESS total
BDI total	*r*	0.426			
	*p*	**<0.001**			
CRI total	*r*	−0.181	−**0.484**		
	*p*	0.037	**<0.001**		
ESS total	*r*	0.087	0.047	−**0.209**	
	*p*	0.319	0.591	**0.016**	

Abbreviations: BDI, Beck's depression inventory; CRI, coping responses inventory; ESS, Epworth sleepiness scale; *P*, test statistic; PSQI, Pittsburgh sleep quality index; *r*, Spearman's rho correlation coefficient. p < 0.05.

### Predictive factors of the Pittsburgh sleep quality index

Multiple linear regression was used to examine the impact of independent factors on the PSQI score; the results showed that the regression results were statistically significant (*F* = 5.477; *p* = 0.001). The PSQI scores were positively impacted by the shift‐work status, BDI score, and ESS score. The PSQI scores were largely unaffected by the BDI and ESS scores, with the status of working shifts having the strongest impact (*p* = 0.024). Additionally, the CRI score had a negligible detrimental impact on the PSQI score.

## DISCUSSION

We found that according to the PSQI scores, 39.8% of the nurses had poor sleep quality, while 25.6% had long‐term sleep disturbances. Khatony et al. found that 77.4% of nurses in the United States had sleep disorders, and Dong et al. reported that 65.8% of nurses in China experienced poor sleep quality (Dong et al., 2020; Khatony et al., 2020). According to the ESS scores, which we applied to evaluate the daytime sleepiness of the nurses, 42.1% of the nurses experienced high daytime sleepiness, while 6.8% experienced severe daytime sleepiness. Akçay et al. reported that 20% of the nurses experienced excessive daytime sleepiness (Akçay et al. 2021) Chen et al. (2019) reported that 16.1% and Gander et al. (2019) reported that 32.5% experienced daytime sleepiness (Chen et al., 2019 Gander et al. 2019) Increased daytime sleepiness is known to reduce alertness among nurses and reduce their performance (contributes to cognitive overload, reduces performance in the activities); it is also associated with twice as many medical errors (Cavalheiri et al., 2021). In addition, we found that the rate of sleep problems was higher in our study sample (65.4%) than in the general population (55.1%) (Duran & Erkin, 2021). The sleep quality of the nurses in our study was lower than that reported in the United States and China (Dong et al., 2020; Khatony et al., 2020) but higher than that in some studies (Deng et al., 2020). The differences in the sleep quality of nurses among different countries may be the result of different working and living conditions.

In our study, it was discovered that the nurses with more working years had significantly better sleep quality than the nurses with fewer working years (*p* < 0.05). These findings imply that newly qualified nurses may experience poorer sleep quality as a result of working in more demanding environments and adjusting to their new careers. Supporting this finding, in a cohort study where the sleep characteristics of new graduate nurses were monitored, it was found that nurses experienced a significant decrease in sleep and quality of life for up to four months after they started working (Kim & Lee, 2022). Insufficient sleep negatively affects nurses' work performance as well as their physical and mental health (e.g., cardiovascular risk, mood disorders, etc.) and patient safety (Chang & Li, 2019). Nurses are responsible for making clinical decisions in the healthcare system. It has been reported in the literature that nurses who have sleep problems increase the likelihood of making mistakes (Abdulah & Suleman, 2021; Cavalheiri et al., 2021; Querstret et al., 2020). Therefore, promoting healthy sleep for nurses is important for both patient safety and their own health.

In our study, the nurses who did not work shifts had considerably higher sleep quality than the nurses who did (*p* < 0.05). In their study on nurses in a hospital in South Korea, Baek et al. (2020) found that sleep patterns varied depending on the type of shift, and sleep deprivation was particularly severe before the day shift. Özvurmaz and Öncü, Akçay et al., and Doğan et al. found that shift work adversely affected the quality of nurses’ sleep, and this circumstance was a matter of concern for the nurses (Akçay et al., 2021; Ozvurmaz et al., 2019). Heath et al. did not discover a statistical correlation between nurses' shift work and sleep quality, in contrast with the findings of our study. They claimed that their results could be attributed to the nurses' familiarity with a continuous shift schedule based on night and day circadian rhythms (Heath et al., 2019). In addition, supporting our research findings, the literature states that shift work causes nurses to constantly change their sleep times and be unable to adapt to their circadian rhythms, resulting in decreased sleep quality and fatigue (Bell et al., 2023; Chang & Li, 2019). In a study by Dires et al. (2023), night shifts affected the work performance of the nurses and increased their workload by 39.3%, fatigue by 30.3%, and concentration problems by 15.6% (Dires et al., 2023). In a meta‐analysis of 38 studies on fatigue in nurses, 82% of the studies identified fatigue as a contributing factor to medication administration errors (Bell et al., 2023). Therefore, sleep problems among nurses working night shifts can not only deteriorate their health and performance but also compromise the quality of care and patient safety (James et al., 2020; Stimpfel et al., 2020). It is important to implement regulations and policies regarding nurses' working hours to ensure safe and quality patient care.

In our study, nurses who did not drink coffee had much better sleep quality than those who did (*p* < 0.05), while nurses who did not drink tea were less likely to take naps during the day. Similarly, Khatony et al. (2020) and Andrijević et al. (2018) found that caffeine use adversely affected the sleep quality of nurses (Khatony et al. (2020) Andrijevit et al. (2018) However, the study of Akçay et al. involving nurses and that of Ergün et al. involving health college students did not find a relationship between coffee consumption and sleep quality, and the studies of Özçelik et al. involving medicine students and Özmen et al. involving undergraduate students did not find a relationship between tea consumption and sleep quality (Akçay et al., 2021; Ergün et al., 2017; Özçelik et al., 2022; Özmen et al., 2022). The disparities between college life and the workplace may be the cause of these discrepancies in the studies. Further research is required to fully understand how tea and coffee consumption affects the quality of one's sleep.

In our study, sleep quality was found to increase as the age of the nurses increased (*p* < 0.005). However, no statistically significant difference was between the ESS scores of nurses by median age (*p* > 0.05). Akçay et al. (2021) and Chaiard et al. (2019) found no statistical difference between the sleep quality of the nurses by age (Akçay et al. 2021; Chaiard et al. 2019). Similar to the results of Karahan et al., we found that the sleep quality of younger nurses was significantly worse, but it improved with increasing age (Karahan et al., 2020). Younger nurses may have poorer sleep quality because they work in busy environments, are on call more frequently, and are less eager to take time off. In addition, older nurses quitting shift work may also be a contributing factor to these patterns.

Studies have reported that nurses are psychologically negatively affected by the difficulties they experience in their professional lives (Costa et al., 2020; Heath et al., 2019; Luceño‐Moreno et al., 2020). In addition, nurses may experience depression as a result of their challenging work environments, where they deal with the pain, suffering, and misery of the patients who depend on them for care (Genç et al., 2022). Dai et al. (2019) reported that nurses working night shifts had higher depression rates, related to poor sleep quality caused by the night shift. Additionally, it has been reported that nurses have the highest level of depression among all healthcare professionals in Turkey (Ceri & Çiçek, 2021); 33.1% of the participants in the study by Segon et al. (2022) showed signs of depression(Segon et al. 2022). In our study, 4.5% of the nurses had severe depression and 42.9% had mild depression. Zengin and Gümüş (2019) found that 68.4% of the nurses had moderate depression (Zengin and Gümüş 2019) Our results concur with those in the literature, demonstrating that nurses experience symptoms of depression and measures are needed to protect them from mental health issues at work.

In our study, nurses with poor sleep quality were found to have mild depression (*p* < 0.05). In a previous study, nurses with depressive symptoms had significantly poorer sleep than those without (Segon et al., 2022). When we examined the impact of independent variables on the PSQI score using multiple linear regression, we found that as the nurses' depression score increased by one unit, their sleep quality index decreased by 0.177 (*p* < 0.05). Similar results have been reported in the literature (Dai, 2019; Salcan & Sarıkaya, 2020). Considering that depressive symptoms indicate a decrease in the amount of serotonin and other neurotransmitters in the brain, depression has been linked to poor sleep quality. Serotonergic neurons have a significant impact on how sleep is initiated and maintained (Keskin & Tamam, 2018). Additionally, further studies are needed to determine the causes of psychological stress in nurses and to reduce the impact of this stress on mental health (such as depression and anxiety).

According to reports, nurses experience professional dissatisfaction and mental health issues due to inadequate compensation for the challenges they face at work (Saquib et al., 2019). Our study revealed that nurses with low socioeconomic status exhibited higher depression scores, increased levels of depression, and decreased inclination to use stress‐reduction techniques (*p* < 0.05). In alignment with our findings, Zengin and Gümüş (2019) reported that nurses with low socioeconomic levels experienced more severe depression. The impact of economic status on the level of depression is further supported by a study conducted by Aslan and Dinç (2022). There is a lack of studies on the connection between nurses' financial status and use of coping mechanisms. Moreover, the use of coping strategies to combat stress plays a pivotal role in mitigating depression and anxiety and maintaining mental well‐being (Ahmad et al., 2022).

## CONCLUSION

In our study, we found that as the nurses' likelihood of using strategies for coping with stress decreased, their depression levels increased and sleep quality decreased. This is an important finding in terms of both the care they provide to the patients and their behaviors in protecting and maintaining their own health. The working conditions of nurses affect their mental state and their likelihood of using strategies to cope with stress, thus affecting their sleep quality. Nurses, especially those who are new to the profession and who work in shifts, need to receive training and consulting services in order to protect and maintain their health. In order to increase the sleep quality of nurses, safeguard their mental health, and increase their potential to use strategies to cope with stress, new plans should be made for the working systems; shift schedules should be prepared in a way to provide adequate rest and reduce daytime sleepiness; appropriate environments should be prepared to enable the nurses to express their problems and improve their personal rights. Additionally, hospital administrations can develop a program for nurses, specifically focusing on coping strategies for stress.

### Implications for nursing practice and policies

In this study, we reported a high prevalence of poor sleep quality among nurses. Factors such as many night shifts, few years of work experience, and coffee consumption were significantly associated with poor sleep among the nurses. Based on the findings of this study, nurses' sleep quality can be improved by implementing stress and mental health management techniques. To improve the working conditions for nurses, new business strategies, and policies should be put into place. Additionally, it is important to adjust working hours and breaks in hospitals to ensure the well‐being of nurses and enhance patient safety. A standard for shift systems in hospitals that remains consistent across different institutions is needed. Hospital administrators should regulate policies regarding nursing employment and working conditions to improve the overall working conditions.

## LIMITATIONS

One limitation of this study is the transferability of the findings to countries with similar shift characteristics. Another limitation is the lack of controlling for other factors that may affect mental health, such as internet usage, social media, family psychiatric history, and so on. Another limitation is the use of a self‐administered questionnaire that relied on subjective measurements for assessing sleep quality.

## AUTHOR CONTRIBUTIONS

*Study design*: GT, AÖ, NGB. *Data collectıon*: GT. *Data analyisis*: GT, AÖ. *Study supervision*: GT, AÖ. *Manuscript writing*: GT, AÖ NGB. *Critical revision for important intellectual content*: GT, AÖ.

## CONFLICT OF INTEREST STATEMENT

The authors declare no conflicts of interest.

## FUNDING INFORMATION

No funding support was received for this study. The authors alone are responsible for the content and writing of this article.
